# What we can learn from hibernators and other adapted animals about organ transplantation: mechanisms of cold resistance and enhanced regeneration

**DOI:** 10.1093/procel/pwag011

**Published:** 2026-03-15

**Authors:** Hannah Esser, Robert J Porte, Luc J W van der Laan

**Affiliations:** Department of Surgery, Erasmus MC Transplant Institute, Erasmus University Medical Center, Rotterdam 3015 GD, The Netherlands; Department of Surgery, Erasmus MC Transplant Institute, Erasmus University Medical Center, Rotterdam 3015 GD, The Netherlands; Department of Surgery, Erasmus MC Transplant Institute, Erasmus University Medical Center, Rotterdam 3015 GD, The Netherlands

Unconventional animal species with specific biological traits provide unique models for scientists searching for inspiration on new therapeutic options. These animal species often show remarkable abilities, such as survival in extreme environmental conditions, which could also be harnessed to combat human diseases. In the field of organ transplantation, researchers are now investigating such unconventional animal models regarding mechanisms of hibernation, super-cooling, and enhanced regeneration to tackle transplant-associated complications and improve outcomes for affected patients.

Organ transplantation is an effective treatment for end-stage organ failure. However, during the transplant process, the organ is subjected to various potentially damaging factors such as cold storage and ischemia-reperfusion injury (Jaeschke, 1996). This is particularly the case for liver transplantation (LT), which often represents the only life-saving treatment for patients with end-stage liver disease or acute liver failure (Samuel et al., 2024). LT is a major procedure and can be associated with the development of post-operative complications. Some of the most frequent and troublesome post-LT complications are caused by transplantation-induced injury of the biliary tract—a system of ducts lined by epithelial cells (cholangiocytes) transporting bile to the digestive tract (Esser et al., 2025). The development of biliary complications has been linked (amongst others) to prolonged cold storage periods (O’Neill et al., 2014). Yet, static cold storage (SCS) still represents the most commonly used “mode of transport” to get the organ from donor to recipient centre. This is despite the increasing use of machine perfusion devices as—due to the complicated logistics of moving the often large machine perfusion devices to the donor centre and back—most transplant centres are applying a back-to-base approach, thus cold-storing the organ during transport and connecting it to a machine perfusion device upon arrival at the recipient centre (Cardini et al., 2020; Eden et al., 2023; Schlegel et al., 2019).

During SCS, livers are subjected to hypoxic conditions at about 4°C. Several studies showed especially the cholangiocytes to suffer profound damage during this SCS period (Brunner et al., 2013; Op den Dries et al., 2014; Esser et al., 2024; Ferreira-Gonzalez et al., 2022). The majority of extrahepatic bile ducts show loss of >50% of the surface epithelium following SCS (Op den Dries et al., 2014). Subsequently, the cholangiocytes need to regenerate and repair the set damage. Failure to repair this damage (= insufficient regeneration) is hypothesized to result in the development of post-operative biliary complications (Esser et al., 2025). Insufficient regeneration following SCS and subsequent LT has been attributed to mitochondrial injury (Eden et al., 2025; Schlegel et al., 2022), damage to the peribiliary glands (Op den Dries et al., 2014; de Jong et al., 2019) or the peribiliary vascular plexus (Tingle et al., 2021; Watson et al., 2023), the induction of cholangiocyte senescence (Esser et al., 2024; Ferreira-Gonzalez et al., 2022), altered electrolyte secretion (and associated collapse of the biliary bicarbonate umbrella) (Roos et al., 2021) and ongoing hypoxia (de Jong et al., 2022). However, most of these mechanisms occur during SCS, leading to insufficient bile duct regeneration and biliary complications after LT. It would therefore be beneficial to identify strategies to minimize damage to the cholangiocytes during the SCS period, hence limiting the damage to biliary epithelium in the first place. Several strategies to minimize cell damage during the SCS period have been evaluated, amongst those supplementing the cold preservation solution with oxygen carriers (Alix et al., 2020) or with agents reducing oxidative stress (Taggart et al., 2025). Some groups also tried adjusting the cold storage temperature to 10°C in order to minimize biliary damage (Tracy et al., 2025). However, although successful in an experimental setting, none of these strategies has made its way to clinical practice.

This lack of therapeutic options to tackle cold stress-related injury researchers has paved the path to working with unconventional animal models in the field of organ transplantation. The utilized unconventional animal models possess remarkable capabilities to deal with cold and/or freezing temperatures. One example from the animal world on how to improve cold resistance comes from hibernating mammals. Hibernators can survive decreased body temperature, suppressed metabolism, and immobility without developing organ injury (de Vrij et al., 2023). Hibernators such as Monito del Monte can spend as long as 6 months in hibernation and slow down their metabolism by about 90% (Fontúrbel et al., 2022). Shrews can even shrink their brain to decrease metabolic demand (Baldoni et al. 2025; Lázaro et al., 2019). Understanding the physiological mechanisms enabling hibernation could improve outcomes in organ transplantation by minimizing organ damage during SCS. In their recent publication in *Protein & Cell*, Wu et al. (2025) investigated whether hibernator-derived cholangiocytes have better cold resistance than cholangiocytes from non-hibernating mice. For this purpose, they used tissue-derived organoid technology (Verstegen et al., 2025) to culture intrahepatic cholangiocyte organoids (ICOs) (Marsee et al., 2021). In a first step, the authors cold-stored livers from the mammalian hibernator Syrian hamster for several days and compared them to livers from normal mice. As reported before, they observed that murine livers display signs of bile duct damage (collapse of the bile duct lumen, epithelial sloughing) after only a few hours of SCS (Ferreira-Gonzalez et al., 2022; Wu et al., 2025). In contrast, cold-stored Syrian hamster livers display preserved biliary architecture even after prolonged periods of SCS and lack the inflammatory response observed in their murine counterparts (Wu et al., 2025). To further investigate these differences in cold resistance in hibernators, the authors cultured both ICOs from Syrian hamsters (shICO) and mice (mICO). When exposed to cold stress shICOs, show improved survival rates compared to mICOs. Further experiments revealed this increased cold resistance to be due to decreased levels of ferroptosis, lipid peroxidation and apoptosis in shICOs (Wu et al., 2025). Similar observations were made with Syrian hamster-derived hepatocytes (Anegawa et al., 2021). While murine hepatocytes subjected to cold temperatures of 4°C undergo cell death within 2 days, Syrian hamster-derived hepatocytes survive 5 days or longer at 4°C. In analogy to Syrian hamster cholangiocytes, the cold resistance in Syrian hamster hepatocytes seems to be mediated by ­differences in ferroptosis and lipid peroxidation (Anegawa et al., 2021). This cross-species comparison between hibernators and non-hibernators could help in identifying novel mechanisms to improve cold resistance in LT; for example, by supplementing the cold storage solution with therapies targeting the pathways mediating the observed cold-resistance. In their article, Wu et al. suggest the use of ferroptosis inhibitors; however, the use of those needs to be carefully evaluated in animal and clinical safety studies before usage in a clinical setting (Wu et al., 2025).

Beyond cold resistance, hibernators also exhibit remarkable metabolic adaptations, such as the ability to tolerate a fatty liver without dysfunction. Despite the rapid hepatic fat storage resulting in fatty livers during the fattening period, hibernators do not show signs of compromised liver function. This seems to be mediated by altered fatty acid metabolization and changes in the gut microbiome (Bao et al., 2023). Following the fattening period, hibernators then rely on metabolizing the build-up lipid storage during the hibernation period, a process that again requires changes in lipid metabolism and transport (Kurtz et al., 2021). This “injury-free lipid accumulation and usage” is especially important in light of the increasing percentage of organ donors with a BMI >30 kg/m^2^ (in the United Kingdom, 31% of all donors now have a BMI >30 kg/m^2^) (NHS Blood and Transplant, 2025). More and more donor livers display some grade of steatosis, which can result in such organs being deemed unsuitable for transplantation. Defatting trials, where the liver is subjected to defatting interventions while on a machine perfusion device, are currently underway to make those organs available for transplantation (Abbas et al., 2024). Understanding how hibernators preserve liver function during rapid fat accumulation and how they then metabolize stored lipids could further improve those efforts to increase the donor pool.

Another example from the animal world on how to improve cold resistance in the area of organ transplantation comes from freeze-tolerant animals. These animals developed a remarkable way to deal with freezing temperatures in their natural habitat, called supercooling. At subzero temperatures, these animals freeze at controlled rates of extracellular ice formation and distribute cryoprotectants to prevent cellular damage (Storey and Storey, 1996). One prominent example to illustrate this supercooling strategy is the Alaskan wood frog. In experimental settings, Alaskan wood frogs can stay frozen at −2.5°C for almost 200 days with a subsequent 100% survival upon thawing. In extracts from their muscles and internal organs a natural antifreeze glycolipid can be found (Larson et al., 2014). Supercooling has recently gained attention in the field of organ transplantation as it has the potential to extend preservation times without exerting a negative effect on organ quality. Supercooling in the setting of organ transplantation refers to ice-free organ preservation at subzero temperatures. de Vries and colleagues published a protocol for supercooling of human livers at −4°C for 20 h (De Vries et al., 2020; De Vries et al., 2019). In a subsequently performed viability assessment using subnormothermic machine perfusion livers showed similar viability parameters before and after supercooling, indicating the feasibility of this approach (De Vries et al., 2019). Thus, supercooling could help in extending liver preservation times and protect the organ during transport.

While strategies from hibernators and freeze-tolerant animals aim to minimize cold-induced injury, enhancing the organ’s innate capacity to repair such injury is equally critical to avoid the development of post-LT complications. Here, the spiny mouse (Acomys)—which has recently drawn attention as a “champion” of mammalian regeneration—offers extraordinary insights into the field of tissue repair. Acomys are small mammals that usually live in arid conditions such as the Middle East, parts of Africa and South East Asia (Gaire et al., 2021; Seifert et al., 2012). Acomys are able to repair large skin wounds without scar formation (Gaire et al., 2021; Tomasso et al., 2023) and can also regenerate whisker pads, including the regrowth of whisker follicles with follicular skeletal muscles and neuronal innervation (Varholick et al., 2025). The increased regenerative capacity of Acomys is not restricted to skin, muscle, and cartilage but also applies to the central nervous system and inner organs. In the setting of severe spinal cord injury, Acomys displays increased axon regeneration alongside decreased levels of reactive astrocytes and can therefore restore spinal cord function and regain hind limb coordination (Nogueira-Rodrigues et al., 2022). During the transplant process, organs are subjected to ischemia-reperfusion injury. Interestingly, Acomys also show an increased resistance towards cardiac and renal ischemia (Okamura et al., 2021; Qi et al., 2016), thus turning Acomys into a promising model for transplant-related research. In the setting of renal ischemia reperfusion injury, Acomys can regenerate tissue without fibrosis or tubular atrophy and—most importantly—can also restore normal renal function (Okamura et al., 2021). Acomys also show decreased inflammation (reduced number of F4/80 macrophages) in the injured tissue when compared to Mus (Okamura et al., 2021). In a model of unilateral renal ischemia reperfusion with subsequent contralateral nephrectomy, Acomys showed normal blood urea nitrogen levels, indicating sufficient renal function, whereas Mus presented with rising blood urea nitrogen levels, indicating progressive renal failure (Okamura et al., 2021).

Isolation of tissue-derived organoids will be a promising approach to further unravel the underlying pro-regenerative pathways in Acomys species. However, to our knowledge, no tissue-derived organoids have been reported and establishing induced pluripotent stem cell cultures has proven difficult (Sandoval et al., 2022).

Studying regeneration-prone animals such as Acomys not only holds the potential to improve outcomes following organ transplantation but also to enhance regeneration *in situ*, therefore, avoiding organ transplantation in the first place.

In summary, unconventional animal models can provide unique insights into biological processes. As organ injury during SCS still remains an unavoidable part of the transplant process, a better understanding of animal survival strategies for cold/freezing temperatures—such as supercooling or hibernation—represents a promising novel approach to develop new therapies (Fig. 1). Tissue-derived organoids (Verstegen et al., 2025), as used by Wu et al. (2025), have been shown to be very useful for inter-species comparisons and to gain new insights for physiological strategies of a wide spectrum of mammalian species, including hibernators. Furthermore, some mammals, such as spiny mice, exert enhanced regenerative capabilities. As biliary complications following LT are linked to insufficient regeneration and an altered wound healing response, insights on how Acomys accomplishes enhanced wound healing and regeneration could open the path for developing new therapeutic strategies to improve clinical outcomes in LT.

**Figure 1.**
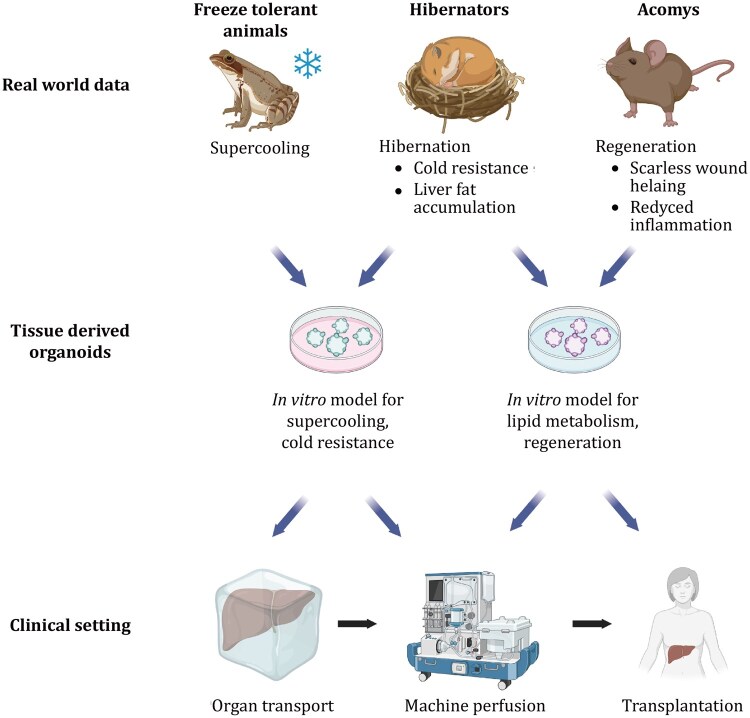
**What we can learn from animal species on cold-resistance and apply to improve outcomes in liver transplantation**. Better understanding of animal strategies on how to survive cold/freezing temperatures could improve outcomes in liver transplantation. Super-cooling as used by freeze-tolerant animals as well as hibernator-inspired cold resistance mechanisms could enhance organ transport and cold storage. Hibernators have an intriguing capacity to deal with fatty livers, a mechanism which could help to de-fat organs on machine perfusion devices and make more organs available for transplantation. Finally, Acomys’ regenerative capacities could be harnessed to boost organ regeneration both during machine perfusion and following transplantation.

## Data Availability

Not applicable.

## Artificial intelligence

No AI-assisted technologies were used to write this article.

## Abbreviations

ICO: intrahepatic cholangiocyte organoid
LT: liver transplantation
mICO: murine ICO
shICO: Syrian hamster ICO
SCS: static cold storage
